# Association of metastatic nodal size with survival in non-surgical non-small cell lung cancer patients: Recommendations for clinical N staging

**DOI:** 10.3389/fonc.2022.990540

**Published:** 2022-10-21

**Authors:** Yanan Zhang, Zhehui Liu, Hongmin Wang, Fengfan Liang, Liqiong Zhu, Haifeng Liu

**Affiliations:** ^1^ Department of Geriatrics, Liaocheng People’s Hospital, Shandong First Medical University, Liaocheng, Shandong, China; ^2^ Joint Laboratory for Translational Medicine Research, Liaocheng People’s Hospital, Shandong First Medical University, Liaocheng, Shandong, China; ^3^ Department of Radiation Oncology, Liaocheng People’s Hospital, Shandong First Medical University, Liaocheng, Shandong, China; ^4^ Department of Radiation Oncology, Shandong Cancer Hospital, Shandong First Medical University, Jinan, Shandong, China

**Keywords:** chemoradiotherapy, non-small cell lung cancer, prognosis, metastatic lymph nodes, staging

## Abstract

**Background:**

This study aims to analyze the prognostic significance of the metastatic lymph node (mLN) size in non-small cell lung cancer (NSCLC) patients receiving chemoradiotherapy (CRT) to provide some information for the optimization of clinical nodal (cN) staging.

**Methods:**

A retrospective study with 325 NSCLC patients was conducted between January 2011 and December 2018 at two participating institutes. We evaluated the potential relationship between the mLN size and the survival to propose a potential revised nodal (rN) staging.

**Results:**

Kaplan–Meier analyses showed significant differences in the overall survival (OS) based on the cN staging and the size of mLNs (N0, ≤2 cm, and >2 cm). We found that the nodal size correlated statistically with the response to CRT. The HRs of OS for patients with bulky mLNs increase significantly compared with patients in the non-bulky mLNs group in the cN2-3 group. Interestingly, the HRs of patients with bulky cN2 disease and non-bulky cN3 disease were similar to each other. We classified the patients into five subsets: N0, rN1(cN1), rN2(non-bulky cN2), rN3a(bulky cN2, and non-bulky cN3), and rN3b(bulky cN3). In our study, the rN stage showed better prognostic discrimination than the 8th IASLC cN staging and was an independent prognostic factor for survival.

**Conclusions:**

In addition to the anatomic location, the size of mLNs correlated statistically with the response to CRT and should be incorporated into the cN staging system to predict survival more accurately.

## Introduction

Lung cancer (LC) is the leading cause of malignant tumor-related deaths worldwide. Non-small cell lung cancer (NSCLC) accounts for 85% of all LC cases (1). Metastatic lymph nodes (mLNs) have a major impact on the survival of NSCLC. Based on a database of 94,708 patients around the world, the 8th edition of the International Association for the Study of Lung Cancer (IASLC) tumor-node-metastasis (TNM) staging for LC was published in 2016 (2). The current N staging for NSCLC depends on the anatomical location of the mLNs and still remains controversial. Many studies have reported the prognostic heterogeneity of patients with the same N staging and the necessity of including other mLN variables in the N staging (3–12). Katsumata et al. demonstrated that the number of mLNs correlated statistically with the patient’s survival (12). In addition, many studies have explored the impact of the mLN ratio on the survival of NSCLC patients who received surgery (13–15). The IASLC staging project also reported that the combination of mLN variables may provide more accurate prognostic information, including the number of mLNs (stations), anatomical location, and lymph node (LN) skipping metastasis (16).

For NSCLC patients who refused to undergo surgery or who were not suitable for surgery, concurrent chemoradiotherapy (CRT) is the standard treatment (17, 18). In contrast to surgical cases, the diagnosis of mLNs in non-surgical patients mostly relies on imaging examinations. The influence of the mLN status on the survival of non-surgical NSCLC is also worth exploring. However, few studies have been done to explore the prognostic effect of the mLNs in NSCLC patients receiving non-surgical treatment. In this study, we analyzed the potential relationship between the mLN size and the prognostic survival of NSCLC patients receiving CRT to provide some information for the optimization of clinical staging.

## Materials and methods

### Patients

We retrospectively analyzed LC patients who received definitive CRT at two independent medical institutions, Shandong Cancer Hospital (Jinan, China) and Liaocheng People’s Hospital (Liaocheng, China), from January 2011 to December 2018. Eligible patients had a pathological diagnosis of non-small cell carcinoma, completion of chemoradiation, and no evidence of distant metastasis imaging before definitive CRT. All patients are evaluated by a thoracic surgeon as surgically unresectable, or refused or unsuitable for surgical treatment for other reasons such as old age or comorbid underlying disease. We excluded patients with dual cancer or survival of less than three months after definitive CRT, and a total of 325 patients met the inclusion criteria. All patients received pre-treatment examinations, including bronchoscopy or ultrasound bronchoscopy, enhanced chest and abdomen computed tomography (CT), and cranial magnetic resonance (MR). Only a small number of patients (131/325) received positron emission tomography-computed tomography (PET-CT) due to economic accessibility. The diagnostic criteria for mLNs were as follows: 1. LNs with a short diameter >1 cm according to CT; 2. LNs with a contrast-enhancing rim or central necrosis. LNs were also considered positive when PET-CT showed a high standardized uptake value (except for inflammatory LNs). We performed endobronchial ultrasound-guided transbronchial needle aspiration (EBUS-TBNA) if necessary to minimize the risk of undetected mLNs (19). The study was approved by the Medical Ethics Committee of Liaocheng People’s Hospital (2021003) and Shandong Cancer Hospital (SDTHEC20190200). Informed consent was exempted due to the retrospective nature of this study. Patient records were anonymized before the analysis of data.

### Treatment

All patients received concurrent CRT that was delivered with three-dimensional conformal radiotherapy (3D-CRT) or intensity modulated radiation therapy (IMRT). The concurrent chemotherapy regimen was platinum-based doublet chemotherapy, and 239 patients received at least one cycle of consolidation chemotherapy. The gross tumor volume (GTV) is defined as the primary lesions or mLNs visible on imaging studies. The clinical target volume (CTV) included the 0.8 cm margin of the primary tumor and the drainage area where the mLNs were located. We outlined the lung, heart, spinal cord, etc. as organs at risk on the CT images and limited the dose. The planning target volume (PTV) was delivered at a total dose of up to 60-66 Gy in 30-33 fractions (5 days a week).

### Follow up

The follow-up of all enrolled patients was up to January 2021. The endpoint of the observation was the overall survival (OS), which is defined as the time from the date of the pathological diagnosis to the date of death. Progression-free survival (PFS) was the duration from treatment date to the date of progression. Failure patterns were defined as: (1) local failure: GTV recurrence including primary tumor and mLNs; (2) distant failure: hematogenous spread to distant organs or non-regional LN metastasis. Efficacy evaluation was carried out one month after chemoradiation, referring to the Response Evaluation Criteria in Solid Tumors (RECIST) Version 1.1 (20). We defined mLNs <15 mm as non-target lesions, and the treatment response was classified as complete response (CR), incomplete response/stable disease (IR/SD), or progressive disease (PD). Primary lung lesions and mLNs ≥15 mm were defined as target lesions, and the treatment response was classified as CR, partial response (PR), SD, or PD.

### Statistical analysis

Fisher’s test was used to determine the difference between groups in the treatment response and different clinical characteristics. We used the Concordance index (C-index) to evaluate the predictive power of different N staging systems. The log-rank test was used for univariate analysis to compare survival differences of patients with different clinicopathological characteristics, and Cox proportional hazard regression model (stepwise backward method) was used for univariate and multivariate analysis to evaluate the potential association between clinical factors and survival. P-values of *p* ≤ 0.05 were considered statistically significant. All statistical analyses were carried out using the SPSS 23.0 (SPSS, Chicago, IL, USA) or the R software version 3.6.1(https://www.r-project.org/).

## Results

### Clinical characteristics

A total of 325 patients consisting of 252 men and 73 women with the median age of 63 years (range, 42-81 years) were enrolled in this study. Among these patients, 222 patients had LN metastasis, and the N2 staging was the most common (35.4%, 114/325), followed by the N0 (31.7%, 103/325), N3 (16.9%, 56/325), and N1 (16.0%, 52/325) staging. Of these patients, 131 underwent EBUS and were pathologically confirmed to have LN metastases. The was median size of the mLNs were 1.6 (range, 1.0-4.3) cm, and 68 patients (20.9%) were classified as T1/2, 183 (56.3%) as T3, and 74 (22.8%) as T4. The incidence of the patients with mLNs ≥1 to ≤2 cm, mLNs >2 to ≤3 cm, and mLNs >3 cm were 44.6% (145 of 325), 16.6% (54 of 325), and 7.1% (23 of 325), respectively. The detailed clinicopathological characteristics are shown in **Table 1**.

**Table 1:** Patient characteristics and univariate analysis of prognostic factors.

Variables	Number of patients (%)	Median OS (months)	*p-*value
Age (years)			0.082
<60	87 (26.8%)	31.0	
≥60	238 (73.2%)	26.7	
Sex			0.618
Male	252 (77.5%)	26.0	
Female	73 (22.5%)	27.6	
Smoking			0.801
Yes	197 (60.6%)	26.5	
No	128 (39.4%)	29.0	
Histologic diagnosis			0.267
Squamous cell	194 (59.7%)	26.0	
Non-squamous cell	131 (40.3%)	33.0	
Clinical stage			< 0.001
II	94 (28.9%)	36.4	
III	231 (71.1%)	23.5	
T staging			< 0.001
T1/2	68 (20.9%)	37.0	
T3	183 (56.3%)	28.2	
T4	74 (22.8%)	18.0	
N staging			< 0.001
N0	103 (31.7%)	39.0	
N1	52 (16.0%)	30.7	
N2	114 (35.4%)	23.0	
N3	56 (16.9%)	13.5	
Size of mLNs			< 0.001
N0	103 (31.7%)	39.0	
≥1 to ≤2cm	145 (44.6%)	26.1	
>2cm	77 (23.7%)	17.3	
Treatment			0.127
IMRT	256 (78.8%)	29.5	
3D-CRT	69 (21.2%)	26.2	

OS, overall survival; mLNs, metastatic lymph nodes; IMRT, intensity modulated radiation therapy; 3D-CRT, three-dimensional conformal radiotherapy.

### Lymph node size and clinical response

**Figure 1:**
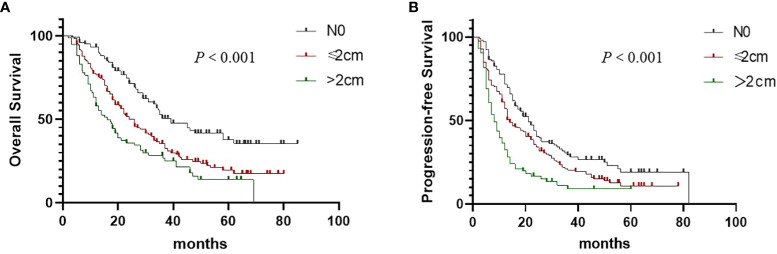
Overall survival **(A)** and Progression-free survival **(B)** in non–small cell lung cancer patients according to the size of metastatic lymph nodes.

In total, 161 patients had distant failure, and 140 had local failure. Moreover, 62 patients had no signs of recurrence or metastasis. We stratified patients with mLNs into three categories according to the size of mLNs as follows: N0, mLNs ≥1 to ≤2 cm, and mLNs >2 cm. Kaplan–Meier analysis showed significant differences in the OS (*P* < 0.001, **Figure 1A**) and PFS (*P* < 0.001, **Figure 1B**) based on the mLN size. The 3-year OS rates were 50.5%, 35.8%, and 26.3%, and the median OS were 39.0, 26.1, and 17.3 months for patients with N0, mLNs ≥1 to ≤2 cm, and mLNs >2 cm, respectively. According to the response to CRT, we stratified all patients into two groups: IR/SD +PD group and PR+CR group. Among all patients, the PR+CR rate was 71.8% in patients with N0, 65.5% in patients with mLNs ≥1 to ≤2 cm, and 61.5% in patients with mLNs >2 cm. As shown in **Table 2**, patients with mLNs >2 cm had the worst treatment response (N0 vs mLNs ≥2 cm: *P =* 0.026, mLNs ≥1 to ≤2 cm vs mLNs >2 cm: *P =* 0.103). The treatment response between N0 groups and mLNs ≥1 to ≤2 cm groups was not statistically different (*P =* 0.292).

**Table 2:** Correlation between the nodal size and the response to treatment.

Groups	PR+CR	IR/SD+PD	Compared groups	*p-*value
N0	74 (71.8%)	29 (28.2%)	≥1 to ≤2 cm	0.292
			>2 cm	0.026
≥1 to ≤ 2cm	95 (65.5%)	50 (34.5%)	>2 cm	0.103
>2 cm	43 (61.5%)	34 (38.5%)		

PR, partial response; CR, complete response; IR/SD, incomplete response/stable disease; PD, progressive disease.

### Current N staging and survival

**Figure 2:**
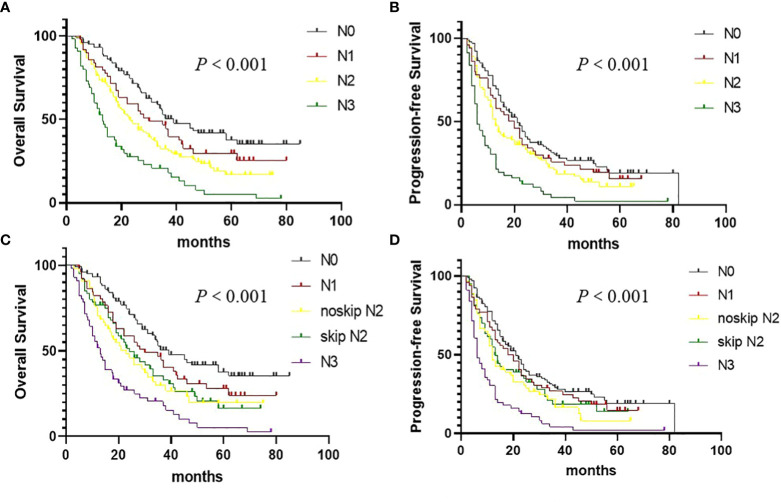
Overall survival (OS) and Progression-free survival in (PFS) non–small cell lung cancer patients according to the cN staging. **(A)** OS curves according to the cN staging. **(B)** PFS curves according to the cN staging. **(C)** OS curves according to the skip cN2 metastasis. **(D)** PFS curves according to the skip cN2 metastasis.

Prognostic factors affecting OS are presented in **Table 1**. For the entire patient group, the N staging depended solely on the anatomical location of mLNs was associated with OS (*P* < 0.001, **Figure 2A**) and PFS (*P* < 0.001, **Figure 2B**) in univariate analysis. The 3-year OS rates were 44.7%, 32.3%, and 18.1%, and the median OS were 30.7, 23.0, and 13.5 months for patients in the cN1, cN2, and cN3 groups, respectively. In addition, we examined the prognostic significance of skip cN2 metastasis on survival (**Figures 2C, D**). We regarded cN2 node metastasis without N1 involvement as skip cN2 metastasis and cN2 node metastasis with cN1 involvement as non-skip cN2 metastasis. In this investigation, skip cN2 metastases were documented in 60 (18.5%, 60/325) patients with cN2 stage NSCLC, and 54 (16.6%, 54/325) patients with cN2 stage NSCLC had non-skip cN2 metastases. Patients with skip cN2 metastases had a better OS than patients with non-skip cN2 metastases, but the difference was not statistically significant (*P =* 0.576, **Figure 2C**).

### Size of metastatic LNs in patients with cN2-3 status: N staging strategy

**Figure 3:**
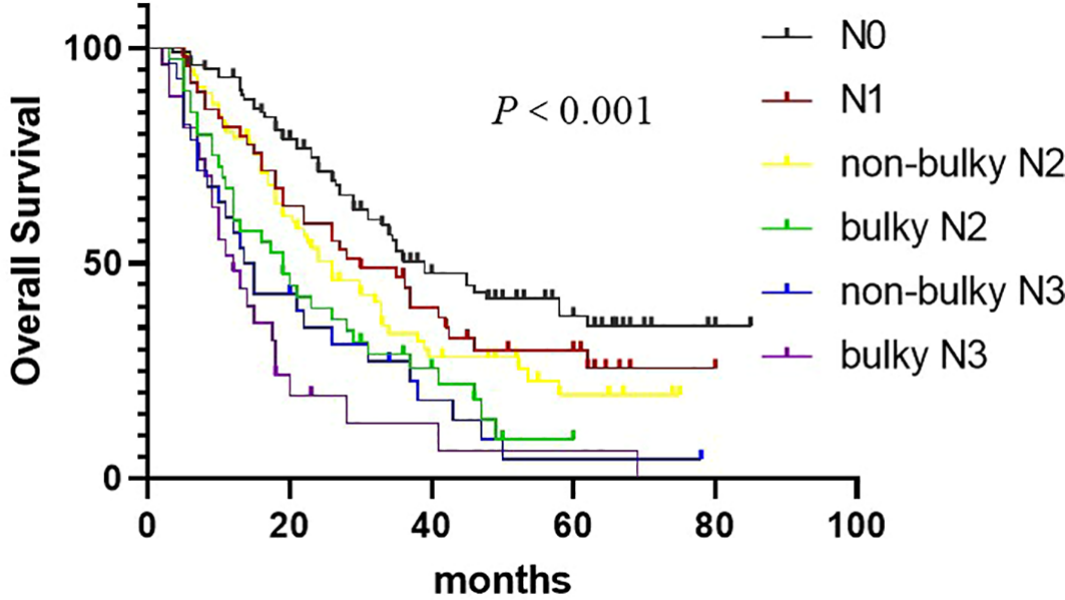
Overall survival in non–small cell lung cancer patients according to the cN subset.

We analyzed the prognostic significance of LN size in patients with cN2-3 Status. Since patients with mLNs >2 cm had a poor response to treatment, and the proportion of patients with 2 cm as the cutoff was reasonable, mLNs >2 cm were defined as bulky LNs in our study. Kaplan–Meier analysis showed patients with bulky LNs had worse OS than those without bulky LNs for patients in the cN2-3 group (**Figure 3**). However, We found no significant differences in OS between N2 patients with bulky mLNs and N3 patients without bulky mLNs (*P* = 0.536). The risk of OS is shown in **Table 3** by the different N subsets (N0, N1, N2 without bulky mLNs, N2 with bulky mLNs, N3 without bulky mLNs, and N3 with bulky mLNs). Multivariate Cox regression analyses showed that the HRs of OS for patients with bulky mLNs increase significantly compared with patients in non-bulky mLNs group. Interestingly, the HRs of patients with bulky cN2 disease (HR, 2.526) and non-bulky cN3 disease (HR, 3.012) were similar to each other (**Table 3**). These results suggest that there is no difference in OS between patients with bulky cN2 disease and patients with non-bulky cN3 disease. So, we propose a potential revised nodal (rN) staging and classified the patients into five subsets: N0, rN1(cN1), rN2(non-bulky cN2), rN3a(bulky cN2, and non-bulky cN3), and rN3b(bulky cN3).

**Table 3:** Univariate and Multivariate Cox regression analysis for the OS of NSCLC patients.

Prognostic Factor	Univariate analysis			Multivariate analysis		
	HR	95% CI	*p-*value	HR	95% CI	*p-*value
Age (baseline: < 60)						
≥60	1.339	0.987-1.818	0.071			NS
Sex (baseline: Male)						
Female	1.077	0.788-1.473	0.641			NS
Smoking (baseline: NO)						
YES	0.960	0.734-1.255	0.764			NS
T staging (baseline: T1/2)						
T3	1.194	0.842-1.695	0.320	1.235	0.864-1.766	0.247
T4	1.874	1.257-2.794	0.002	1.846	1.229-2.775	0.003
cN subset (baseline: N0)						
N1	1.428	0.938-2.174	0.097	1.479	0.970-2.255	0.069
N2 without bulky mLNs	1.641	1.115-2.415	0.012	1.600	1.081-2.367	0.019
N2 with bulky mLNs	2.540	1.652-3.905	< 0.001	2.526	1.576-3.736	< 0.001
N3 without bulky mLNs	2.958	1.840-4.755	< 0.001	3.012	1.860-4.877	< 0.001
N3 with bulky mLNs	4.412	2.710-7.184	< 0.001	4.173	2.551-6.828	< 0.001

OS, overall survival; HR, hazard ratio; CI, confidence interval; mLNs, metastatic lymph nodes.

**Figure 4:**
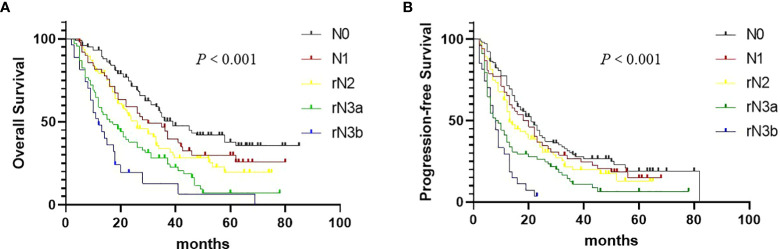
Overall survival **(A)** and Progression-free survival **(B)** in non–small cell lung cancer patients according to the rN staging.

We found OS (*P* < 0.001, **Figure 4A**) and PFS (*P* < 0.001, **Figure 4B**) were significantly correlated with the rN staging. In our study, rN stage (C index: 0.625, 95% CI: 0.574-0.676) showed the better prognostic discrimination than 8th IASLC N staging(C index: 0.616, 95% CI: 0.563-0.667). In combination with T stage, rN stage was also superior to 8th IASLC N staging(C index: 0.654, 95% CI: 0.603-0.705 VS 0.633, 95% CI: 0.581-0.687; **Table 4**). **Table 5** presents the results of the multivariate analyses of OS, local failure, and distant failure. In the current study, rN stage was an independent prognostic factor for all.

**Table 4:** Comparison of the C index of different staging systems.

Staging systems	C index	95% CI
rN staging	0.625	0.574-0.676
rN staging +T staging	0.654	0.603-0.705
IASLC cN staging	0.616	0.563-0.667
IASLC cN staging +T staging	0.633	0.581-0.687

CI, confidence interval; rN staging, revised nodal staging.

**Table 5:** Effect of rN staging on risk of death, local failure, and distant failure.

Prognostic Factor	Death			Local failure			Distant failure		
	HR	95% CI	*p*-value	HR	95% CI	*p*-value	HR	95% CI	*p*-value
Age									
<60	1			1			1		
≥60	1.248	0.911- 1.709	0.168	1.134	0.763-1.685	0.534	1.206	0.831-1.751	0.324
Sex									
Male	1			1			1		
Female	1.098	0.796-1.516	0.568	0.877	0.573-1.343	0.547	1.021	0.831-1.744	0.326
Smoking									
Yes	1			1			1		
No	0.848	0.606-1.187	0.337	1.003	0.655-1.534	0.991	1.126	0.818-1.549	0.468
T staging									
T1/2	1			1			1		
T3	1.235	0.864-1.766	0.247	1.149	0.744-1.774	0.532	1.210	0.806-1.817	0.357
T4	1.846	1.229-2.775	0.003	1.550	0.932-2.577	0.089	1.836	1.147-2.940	0.011
rN staging									
N0	1			1			1		
rN1	1.497	0.974-2.303	0.066	1.112	0.654-1.891	0.694	0.945	0.601-1.486	0.808
rN2	1.601	1.089-2.454	0.017	1.331	0.843-2.103	0.220	1.107	0.687-1.784	0.677
rN3a	2.467	1.608-3.785	< 0.001	1.941	1.129-3.336	0.016	1.697	1.014-2.842	0.044
rN3b	3.389	2.278-5.040	< 0.001	2.332	1.395-3.898	0.001	2.856	1.829-4.461	< 0.001

HR, hazard ratio; CI, confidence interval; rN staging, revised nodal staging.

## Discussion

The mLN variables have attracted not only the special attention of surgeons but also of radiation oncologists because the location of mLNs is an essential factor in determining the CTV of radiotherapy. However, the data of the IASLC includes a variety of treatment methods, while the prognostic significance of the N stage in NSCLC patients receiving definitive radiotherapy is not specified (2). To evaluate the prognostic significance of metastatic LN variables in non-surgical LC, we systematically reviewed NSCLC data from two independent medical institutions. Our data show that a combination of size and anatomic location of metastatic LNs in non-surgical ESCC patients leads to good accuracy and stratification ability.

In contrast to the pathological staging, clinical staging is mainly based on imaging examinations, and the most commonly used clinical imaging examinations are ultrasound bronchoscopy, CT, and PET-CT. Accurate imaging-based staging of NSCLC patients has an important influence on the choice of the treatment strategy and the assessment of prognosis. Compared with PET-CT, PET-MR showed similar diagnostic performance for N staging of NSCLC patients (21). A study showed that the accuracy of the N staging is 68% for PET, and 63% for CT (22). In addition, the lung is the only tumor location where the N staging is determined only by the location, regardless of the tumor burden of mLNs. In the current clinical staging based on location, the tumor burden of regional mLNs is not fully reflected. For example, a single small mLN, metastases visible under a microscope, the involvement of mLNs with a size of 2 cm, or multiple mLNs at the same location belong to the same N category. Therefore, it is necessary to analyze the impact of multiple mLN variables on the survival to assess the prognosis of patients more accurately.

The number of mLNs is currently used in the N staging of multiple solid tumors, such as breast, head and neck, and digestive tract tumors (23). Many studies have reported the necessity of including the number of mLNs in the N staging of NSCLC (3–8). Saji et al. analyzed 689 NSCLC patients undergoing surgical treatment to develop a revised pathological N staging based on the combination of the number and location of mLNs. They found that this N staging was more accurate than the pathological N staging to predict prognosis (7). Shang et al. screened 9539 resected NSCLC patients from the SEER database and came to similar conclusions (24). Many studies also investigated the prognostic impacts of the LN ratio of mLNs on the survival of resected NSCLC (13–15). Ding et al. reviewed 700 patients with prognostically heterogeneous pN1 or pN2 NSCLC and proposed a revised pN staging integrating the LN ratio and the anatomic location of mLNs to further stratify patients into subgroups and to predict prognosis more precisely (13). However, these results provide limited information for non-surgical patients. The coincidence rate of LN metastasis between preoperative clinical staging and postoperative pathological staging is poor, and the number of LNs, such as clustered or clumpy LNs, is difficult to determine depends solely on imaging diagnosis.

Few studies have been done to explore how the size of LNs influences the survival in NSCLC patients treated with definitive CRT. The size of LNs is the main diagnostic criterion for the imaging examination of mLNs. Previous studies have shown that bulky mLNs are associated with the survival of surgical NSCLC patients with pN2 disease but not with the survival of NSCLC patients with pN1 disease (25, 26). In addition, studies have reported that bulky mLNs are significantly related to LN extravasation (27, 28). So, the size of mLNs has an important prognostic value in the N staging of head and neck cancer. Shih BC et al. reviewed 282 pathologic stage III-N2 NSCLC patients and found that the presence of LN extravasation is associated with poorer survival (29). The same conclusion was also drawn in Nomura K et al. ‘s study (30). Our results show that the size of mLNs is a prognostic factor for non-surgical NSCLC patients and is related to the treatment response. Non-bulky LNs respond better to treatment, which may account for their favorable prognosis. Considering the above results, we believe that incorporating LN size into clinical staging is beneficial to improve the prediction accuracy of cN staging.

However, this study has the following limitations. Due to its retrospective nature, some information, such as the histological grade, was not available in all cases. Second, although we analyzed data from two independent medical institutions, the small sample size may not be very reliable. Further large sample studies based on non-surgical datasets are needed. Third, the heterogeneity of the treatment, such as the types of additional therapies after recurrence, may affect the results. However, the value of this study lies in the analysis of the impact of the size of mLNs on non-surgical patients, which has not been reported in previous studies. Our results provide reference for the optimization of lung cancer clinical staging in the future.

## Conclusions

The size of mLN, as well as the anatomic location of mLN, has an impact on the survival of non-surgical NSCLC patients. Our results show that incorporating mLN size into clinical staging improves the accuracy of cN staging. These results also provide some information on the future staging system of NSCLC. However, our results need to be verified by a prospective, multi-institutional study.

## Data availability statement

The raw data supporting the conclusions of this article will be made available by the authors, without undue reservation.

## Ethics statement

The study was approved by the Medical Ethics Committee of Liaocheng People’s Hospital (2021003) and Shandong Cancer Hospital (SDTHEC20190200). Informed consent was exempted due to the retrospective nature of this study.

## Author contributions

YZ: conceptualization. formal analysis. investigation. resources. writing – original draft. and approval of the manuscript. ZL: conceptualization. data curation. formal analysis. methodology. visualization. writing – original draft. and approval of the manuscript. HW: formal analysis. investigation. and approval of the manuscript. FL: conceptualization. data curation. investigation. resources. and approval of the manuscript. LZ: data curation. investigation. resources. and approval of the manuscript. HL: conceptualization. data curation. methodology. supervision. writing – original draft. writing – review & editing. and approval of the manuscript. All authors contributed to the article and approved the submitted version.

## Funding

This study was funded by the Natural Science Foundation of China (NSFC 81672995).

## Conflict of interest

The authors declare that the research was conducted in the absence of any commercial or financial relationships that could be construed as a potential conflict of interest.

## Publisher’s note

All claims expressed in this article are solely those of the authors and do not necessarily represent those of their affiliated organizations, or those of the publisher, the editors and the reviewers. Any product that may be evaluated in this article, or claim that may be made by its manufacturer, is not guaranteed or endorsed by the publisher.
